# Use of Ceftolozane-Tazobactam in Patient with Severe Medium Chronic Purulent Otitis by XDR *Pseudomonas aeruginosa*

**DOI:** 10.1155/2019/2683701

**Published:** 2019-10-09

**Authors:** L. M. Saraca, C. Di Giuli, F. Sicari, G. Priante, F. Lavagna, D. Francisci

**Affiliations:** ^1^Clinica di Malattie Infettive, Università degli Studi di Perugia, A. O. “S. Maria”, Terni, Italy; ^2^Clinica di Malattie Infettive, A. O. “S. Maria”, Terni, Italy; ^3^Struttura Complessa di Radiologia, A. O. “S. Maria”, Terni, Italy

## Abstract

We present a case of a male Italian patient of 66 years with a history of kidney transplantation in treatment with cyclosporine and methylprednisolone. He visited an ENT clinic and was diagnosed as chronic left purulent otitis media. He began at-home antibiotic therapy with poor benefit. On 09/13/18, he was admitted to the hospital “S. Maria ”of Terni for persistence of left ear pain and complete hearing loss. Magnetic resonance imaging (MRI) of the brain showed “*in correspondence of the petrous rock and the mastoid…presence of flogistic tissue*.” Auricular swabs and later surgical drainage of the purulent material were performed and both were positive for extensively drug-resistant (XDR) *Pseudomonas aeruginosa* sensitive only to colistin in absence of synergism with rifampin. The patient underwent antibiotic therapy with ceftolozane-tazobactam, a new generation cephalosporin with anti-Pseudomonas activity and a *β-*lactamase inhibitor, that currently is indicated for the treatment of complicated urinary tract infections and complicated intra-abdominal infections, with complete healing. In literature, it is described a series of 12 patients with severe MDR (multidrug-resistant) *Pseudomonas aeruginosa* infections (6 pneumonia) who received salvage therapy with ceftolozane-tazobactam after inappropriate empirical and/or suboptimal treatment. This study included a case of a male patient of 45 years, affected by Burkitt lymphoma and severe neutropenia, who presented with otitis and mastoiditis, and isolation of *Pseudomonas aeruginosa* in surgical drainage of the purulent material of the ear (blood cultures were negative). He underwent antibiotic therapy with ceftolozane–tazobactam at a dosage of 3 g/8 h for 21.3 days. The patient was healed, but a late recurrence was described because of isolation of ceftolozane-tazobactam-resistant *Pseudomonas* after therapy. The possibility of acquiring resistance to ceftolozane-tazobactam should be considered in patients with previous exposure to beta-lactams and with poor response to these antibiotics.

## 1. Introduction

*Pseudomonas aeruginosa* is a Gram-negative aerobic bacillus, frequently multidrug resistant, which can cause generalized severe infections, especially in immunosuppressed patients, or localized to an organ [1]. *P. aeruginosa* ear infections can be classified into simple external otitis (or swimmer's ear), malignant outer otitis, otitis media, and perichondritis. Otitis media, in particular, is an inflammation of the middle ear that mainly affects the pediatric population and can be classified as acute and chronic [2, 3]. The acute form is characterized by the rapid onset of local and systemic symptoms as follows: otalgia, hypacusia, feeling of “encumbrance” by accumulation of secretion in the middle ear, fever, nausea, and vomiting [4]. Acute media otitis may also occur in the recurrent form, 3 episodes in six months or 4 in twelve and may evolve in 30–40% of cases in the chronic purulent form, characterized by protracted inflammation, thickening of the middle ear mucosa, perivascular fibrosis, osteitis of the tympanic and mastoid cells, persistent otorrhea, possible perforation of the eardrum, and deafness [5–7]. *Streptococcus pneumoniae, Haemophilus influenzae*, and *Moraxella catarrhalis* are the pathogens most frequently causing acute otitis [8–10]. *Pseudomonas aeruginosa* and *Staphylococcus aureus* are the pathogens most commonly associated with the chronic form. Fungi or anaerobic bacteria such as *Clostridium* spp., *Peptococcus* spp., *Prevotella melaninogenica*, and *Fusobacterium* spp. may play a role in the genesis of chronic purulent media otitis. Viruses are not a direct cause of otitis media, but can predispose to infections of the middle ear causing inflammation at the nasopharyngeal level with consequent involvement and alteration of the physiological emptying of the eustachian tube. *Influenza A* virus, coronaviruses, and *respiratory syndrome virus* are often a cause of otitis media [11–13]. Vaccination is considered the main measure to reduce the incidence of otitis. First-line antibiotic therapy for otitis media is amoxicillin, often associated with a *β-*lactamase inhibitor, or 2nd- and 3rd-generation cephalosporins or macrolides, particularly in patients with allergies to penicillins [12, 13]. Ceftolozane-tazobactam is a 2 : 1 coformulation of a new-generation cephalosporin with anti-Pseudomonas activity and a *β-*lactamase inhibitor. It currently is indicated for the treatment of complicated urinary tract infections (UTI) and complicated intra-abdominal infections (IAI), the latter in association with metronidazole. The drug is active against *Pseudomonas aeruginosa*, also against multidrug-resistant (MDR) or extensively drug-resistant (XDR) *Pseudomonas,* and represents a valid alternative to carbapenems for *Escherichia coli* and *Klebsiella* producing broad-spectrum *β-*lactamases [14, 15]. Its antimicrobial activity makes it a suitable and attractive option for off-label treatment of different infections. A multicenter observational retrospective real-world study of 101 hospitalized patients in 22 public Italian hospitals, who were treated for *P. aeruginosa* infections between June 2016 and March 2018, describes the use of ceftolozane-tazobactam to treat infections such as nosocomial pneumonia, acute bacterial skin and skin structure infections (ABSSSI), complicated UTI, complicated IAI, bone infections, and primary bacteraemia. Almost half of *Pseudomonas aeruginosa* strains in the study were XDR (51%) with 78% of the isolates resistant to at least one carbapenem, and ceftolozane-tazobactam was used as first-line therapy in 39 patients (34.6%) at a dosage of 1.5 g/8 h in 70 patients and an off-label dosage of 3 g/8 h in 31 patients; the median duration of therapy was 14 days. The overall clinical success was 83.2%, in particular in respiratory tract infections treated at an antibiotic dosage of 3 g/8 h, and significant lower success rates were observed in patients with sepsis or those receiving continuous renal replacement therapy [16]. Ceftolozane inhibits all PBPs produced by *P. aeruginosa* and *E. coli*. It shows greater activity against *P. aeruginosa* than most *β*-lactams as it is more resistant to the AmpC enzymes produced by the microorganism, and it is not susceptible to active extrusion and it is not significantly affected by porin changes [17]. Ceftolozane-tazobactam has been shown to have dose-dependent pharmacokinetics in healthy adults, not significantly influenced by age, gender, ethnicity, or liver function. Both ceftolozane and tazobactam are eliminated mainly as unchanged molecules in urine: in consideration of the mainly renal excretion, the pharmacokinetics is significantly altered in patients with moderate and severe renal insufficiency. The risk of drug interactions relevant for ceftolozane/tazobactam is low (in vivo studies indicate that the drug is not a substrate for CYP enzymes). After single and multiple administrations every 8 h of 1.5 g of the drug (1 g of eftolozane + 0.5 g of azobactam), maximum plasma concentrations of ceftolozane of 69.1 *μ*g/mL and 74.4 *μ*g/mL, respectively, were detected. Ceftolozane and tazobactam are bound to plasma proteins for 16–21% and 30%, respectively [18–21].

## 2. Clinical Case

**Figure 1:**
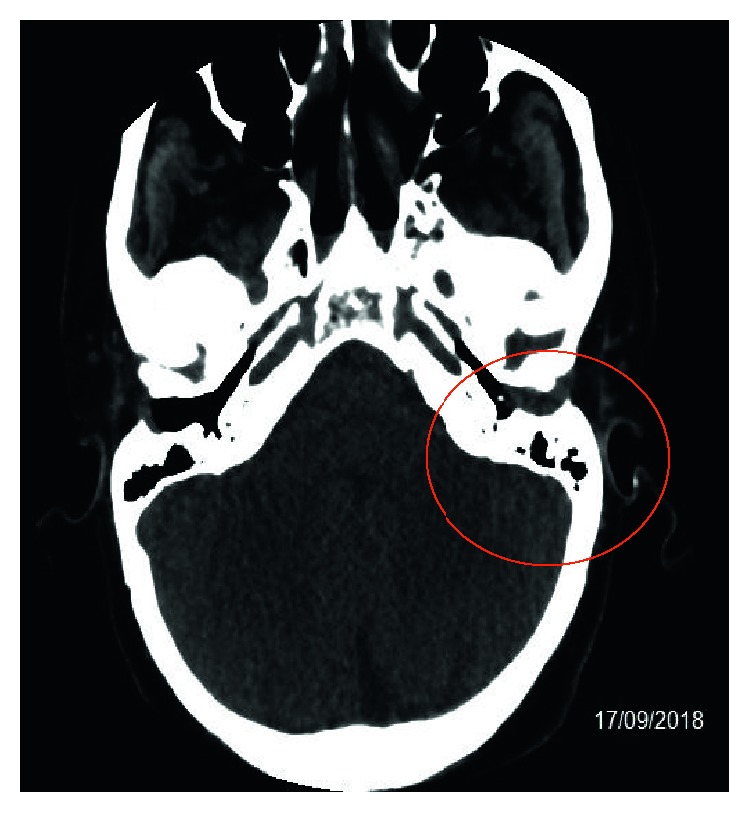
CT of the left ear without contrast medium.

A male Italian patient of 66 years presented with a history of kidney transplantation in treatment with cyclosporine, intestinal resection for lymphoma, and arterial hypertension and heart failure; in July 2018, he visited an ENT clinic and was diagnosed as chronic left purulent otitis media and began at-home antibiotic therapy with poor benefit. On 13/09, he was admitted to the hospital “S. Maria” of Terni for persistence of left ear pain and complete hearing loss in the absence of fever or other symptoms. A computerized tomography (CT) of the left ear without contrast medium documented “*thickening of the walls of the external auditive canal, marked thickening of the tympanic membrane*, *and the ossicular chain conglobated by inflammatory material*” (Figure 1).

**Figure 2:**
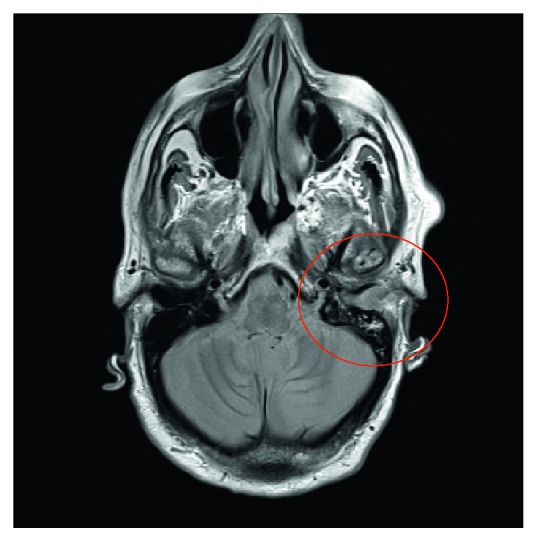
Brain MRI without and with contrast medium.

Thereafter, antibiotic therapy with ertapenem was administered once daily in the day-ospital regimen, and it continued for eight days with no benefit because of persistent pain and hearing loss of the left ear. The blood exams showed nonsignificant values of C-reactive protein and white blood cells (WBC) because the patient took immunosuppressive therapy. Then, on 28/09, the patient was hospitalized at our department, and a brain MRI without and with contrast medium showed “*in correspondence of the petrous rock and the mastoid on the left*, *a nonhomogeneous hyperintensity in the long TR sequences, compatible with the presence of flogistic tissue*” (Figure 2).

**Figure 3:**
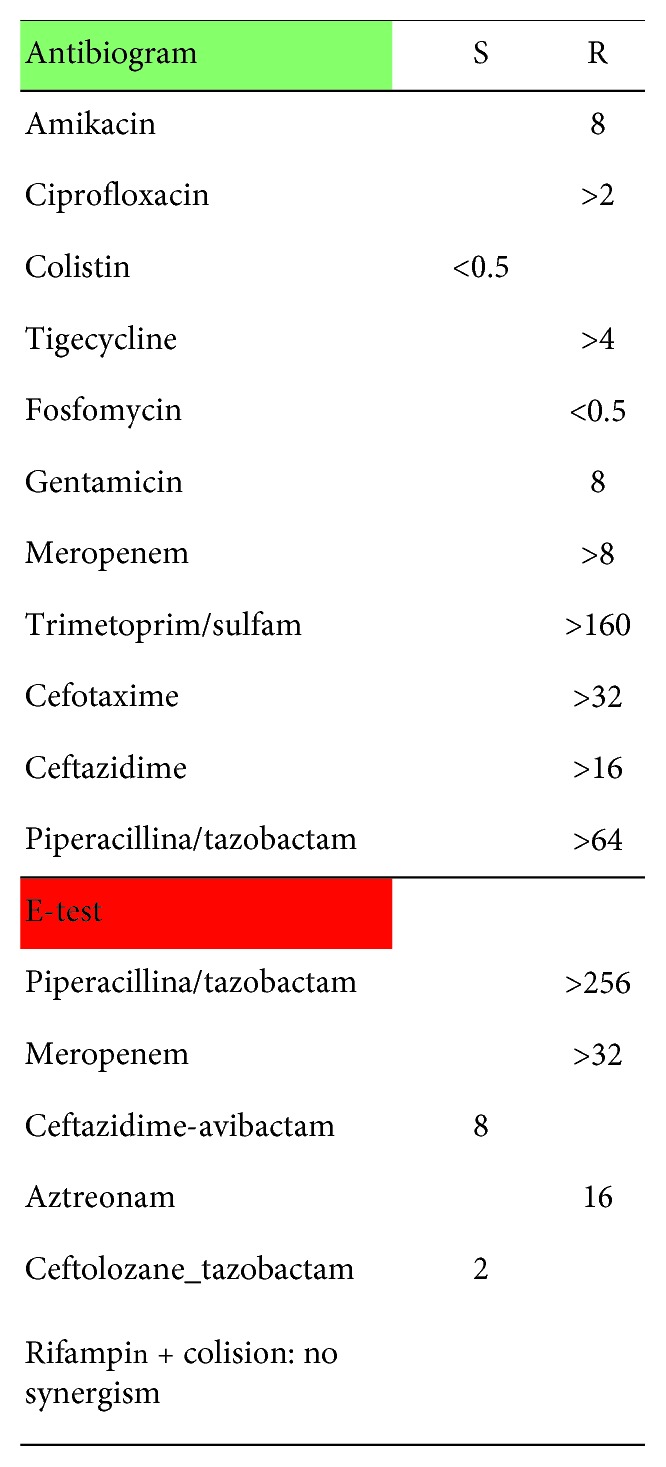
Antibiogram of XDR *Pseudomonas*.

During admission, he was initially treated with empiric intravenous antibiotic therapy with imipenem 500 mg/8 h and linezolid 600 mg/12 h. Blood and urine cultures were negative. Auricular swabs and later surgical drainage of the purulent material present in the left ear were performed and both were positive for XDR *Pseudomonas aeruginosa*, sensitive only to colistin in absence of synergism with rifampicin. On the isolated strain, E- test was performed for ceftolozane-tazobactam and ceftazidime-avibactam with MIC 8 and 2, respectively (Figure 3). 

**Figure 4:**
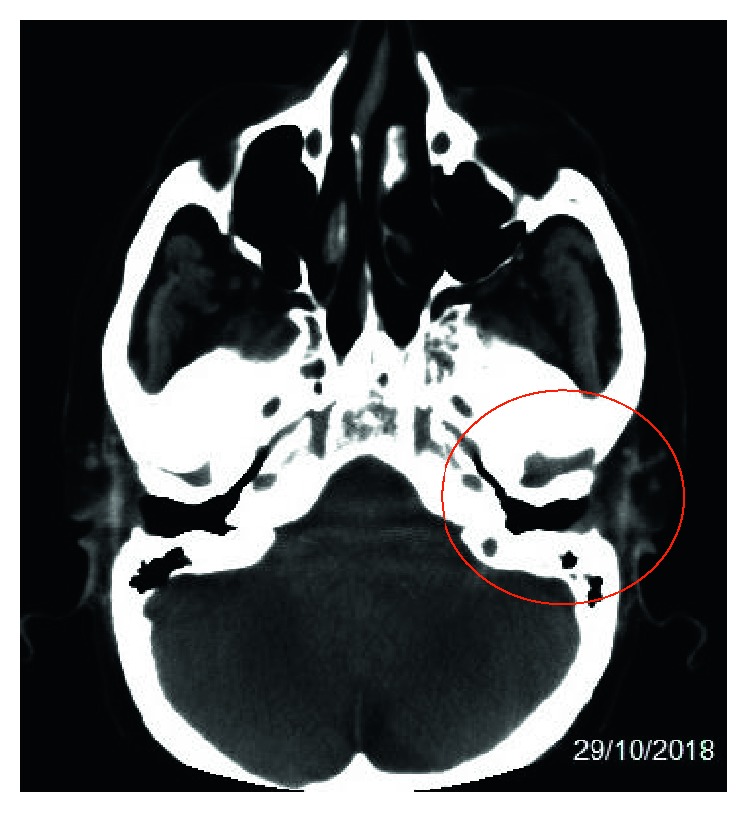
CT scan of the left ear without contrast.

The blood cultures were negative for *Pseudomonas aeruginosa*. From 17/10 to 02/11, the day of discharge, the patient underwent antibiotic therapy with ceftolozane-tazobactam at a dosage of 0.75 g/8 h for the first 4 days and, after abundant hydration and improvement in renal functionality at the dosage of 1.5 g/8 h. The patient presented a progressive clinical improvement with the disappearance of the ear pain and slow resumption of auditory function. The CT scan of the ear without contrast, performed on 10/29/2018, confirmed a “*significant reduction of inflammatory material occupying the middle ear and reduction of thickening of the tissues of the external auditive canal.*” (Figure 4).

## 3. Conclusions

Ceftolozane-tazobactam may represent an important resource against MDR and XDR *Pseudomonas aeruginosa* for the treatment of complicated urinary tract infections (UTI) and complicated intra-abdominal infections (IAI) in accordance with the data of literature, but in the future also in other types of infection like our case or respiratory tract infection.
